# Rhizosphere Microbial and Biogeochemical Differences Associated with Root Rot Occurrence in *Aucklandia lappa*

**DOI:** 10.3390/microorganisms14092049

**Published:** 2026-09-14

**Authors:** Zhen-Huai Fan, Zheng-Qin Su, Rong-Chan Li, Wei Di, Xian Wang, Xian Yang, Bao-Li Qiu

**Affiliations:** 1 Engineering Research Center of Biotechnology for Active Substances, Ministry of Education, College of Life Sciences, Chongqing Normal University, Chongqing 401331, China; zhenhuaifan0820@163.com (Z.-H.F.);; 2 Chongqing-Rwanda Belt and Road Joint Laboratory for Integrated Pest Management of Fruits, Vegetables and Grains, Chongqing Normal University, Chongqing 401331, China

**Keywords:** *Aucklandia lappa*, microbial community, root rot, soil physicochemical properties, enzyme activity

## Abstract

*Aucklandia lappa* Decne is a valuable medicinal herb highly susceptible to root rot, a disease that severely affects its yield and quality. This study provides a comprehensive characterization of the soil ecosystems in *A. lappa* habitats by integrating biogeochemical and microbial functional analyses. Rhizosphere soil samples were collected from healthy plants growing in loam soil (HLS), root-rot-affected plants growing in loam soil (RRLS), and healthy plants growing in clay soil (HCS) from habitats in the Kaizhou District of Chongqing, China. We analyzed soil physicochemical properties (pH, available nitrogen, potassium, phosphorus, organic matter content, and total carbon), enzyme activities (amylase, cellulase, saccharase, β-glucosidase, β-xylosidase, and laccase), and microbial community structure (using 16S/ITS sequencing). The results demonstrated significant differences in nutrient levels and enzyme activities between healthy and diseased soils, with root-rot-affected soils displaying higher nutrient content and distinct microbial diversity. Specific bacterial (*Rahnella*) and fungal genera (*Trichosporiella*, *Cyberlindnera*, *Barnettozyma*, and *Ilyonectria*) were enriched in root-rot-associated soils. This suggests that these microorganisms may participate in rhizosphere ecological responses associated with root rot occurrence, although their roles may include pathogen-associated processes or potential plant adaptation responses. The results demonstrate that root rot occurrence in *A. lappa* is associated with coordinated changes in rhizosphere nutrient status, enzyme activities, microbial community composition, and predicted functional profiles. However, the observational nature of this study does not allow causal relationships between these changes and root rot development to be established.

## 1. Introduction

*Aucklandia lappa* Decne, a perennial medicinal plant of the Asteraceae family, is widely cultivated in southwestern China, particularly in Chongqing, Yunnan, and Sichuan provinces [1,2]. Its roots are valued for their pharmacologically active sesquiterpene lactones, particularly costunolide and dehydrocostus lactone [3,4,5]. However, root rot represents an important constraint on *A. lappa* cultivation and can substantially impair root quality and plant survival [6]. *A. lappa* is commonly cultivated in cool, high-altitude environments. Root rot symptoms include progressive root discoloration, water-soaked lesions, tissue decay, and, in severe cases, plant wilting and death [6]. Root rot diseases in medicinal plants are commonly associated with soil-borne pathogens, including *Fusarium* spp., *Rhizoctonia solani*, and *Ilyonectria* spp., which have been reported to cause severe root diseases in crops and medicinal plants. In *A. lappa*, although several fungal pathogens have been associated with root rot symptoms, the ecological interactions among pathogens, soil properties, and microbial communities remain poorly understood. Previous studies have indicated a strong association between the incidence of tobacco root rot and soil microorganisms, with low abundance of Rubrobacter and Talaromyces potentially linked to the disease [7]. Similarly, microorganisms have been explored as biocontrol agents against root rot in soybeans [8].

In China, *A. lappa* is widely cultivated in the mountainous regions of Kaizhou District, Chongqing. The region is characterized by steep terrain, relatively thin soil layers, abundant precipitation, and substantial spatial variation in soil properties. These environmental conditions may influence nutrient availability, rhizosphere microbial communities, and the occurrence of soil-borne diseases. Therefore, understanding the relationships among soil physicochemical properties, extracellular enzyme activities, and rhizosphere microbial communities is important for evaluating the soil ecological conditions associated with *A. lappa* cultivation and root rot occurrence [9].

Soil pH exerts a profound influence on biogeochemical processes and is often regarded as the “master soil variable” that governs numerous biological, chemical, and physical properties affecting plant growth and biomass yield [10,11]. Nitrogen, phosphorus, and potassium are essential macronutrients for plant development. Nitrogen, predominantly present in organic forms in soil, is a key component of proteins, nucleic acids, and chlorophyll, directly influencing plant growth and yield [12]. Phosphorus, though less abundant and mainly inorganic, promotes early root development and enhances plant adaptability to environmental stress, including cold tolerance—a critical trait for alpine plants like *A. lappa*, which endures much rain and snow from October to May [13]. Potassium, abundant in ionic form, improves water uptake and retention, thereby strengthening plant resistance to drought and cold stress [14]. Total carbon in soil plays a crucial role in plant growth, the synthesis of bioactive compounds, photosynthesis, and the tricarboxylic acid cycle [15,16]. Soil organic matter is vital for maintaining soil quality and productivity, with microbial and enzymatic activities significantly influencing its turnover and storage [17,18,19].

Soil nutrient deficiencies and pathogen invasions pose major threats to crops. Soil microorganisms can enhance plant nutrition and growth, and certain root-associated bacteria and fungi secrete compounds that suppress pests and pathogens [20,21]. In the rhizosphere, bacteria represent one of the most abundant and diverse microbial groups. They are essential for nutrient cycling, including nitrogen fixation, phosphorus solubilization, and potassium mobilization, and also for the synthesis of phytohormones and enzymes that suppress diseases and stimulate plant growth [22,23]. Fungi are another integral component of the soil ecosystem: symbiotic mycorrhizal fungi facilitate water and nutrient uptake by plant roots, while free-living fungi decompose organic matter and contribute to nutrient recycling [24].

This study aimed to elucidate the coupling relationships among soil physicochemical properties, enzyme activities, and microbial diversity (assessed via 16S and ITS sequencing) in *A. lappa* habitats. We investigated the following: (i) whether root rot status was associated with differences in soil nutrient availability and carbon-cycle-related enzyme activities; (ii) whether bacterial and fungal diversity and community composition differed between healthy and root-rot-affected loam rhizospheres; and (iii) whether predicted microbial functional profiles were associated with root rot status and measured soil properties. The findings are expected to provide insights into rhizosphere ecological changes associated with root rot occurrence, improving our understanding of plant–soil–microbe interactions in *A. lappa* cultivation systems.

## 2. Materials and Methods

### 2.1. Sample Collection

Soil samples were collected from the main production area of *A. lappa* in Kaizhou District, Chongqing, China (108.723076–108.772229° E, 31.503904–31.515099° N) (Table S1). The predominant soil type in this region is loam. Sampling points were selected following an S-shaped field sampling scheme to obtain spatially distributed samples within each sampling area. Three separate sampling points were used as biological replicates for each soil category. The geographic coordinates and soil-texture information for each sampling point are provided in Table S1. Sampling points belonging to the same category were located within a relatively small geographic area. For each sampling point, both the root system and rhizosphere soil of *A. lappa* were collected to facilitate accurate separation of rhizosphere soil in subsequent processing. All samples were immediately stored on dry ice. Three types of rhizosphere soils were collected: healthy loam soil (HLS), root-rot-affected loam soil (RRLS), and healthy clay soil (HCS). Differences were mainly compared between HLS and HCS, HLS and RRLS, but not between RRLS and HCS, since RRLS and HCS differ simultaneously in both disease status and soil texture; direct comparisons between these groups would confound the interpretation of disease- versus soil texture-driven effects. Therefore, our analyses primarily focused on (i) HLS vs. RRLS to evaluate root-rot-associated changes within the same soil type and on (ii) HLS vs. HCS to assess the differences between healthy loam and healthy clay rhizosphere soils.

Three biological replicates were collected for each soil category (HLS, RRLS, and HCS), resulting in 18 independent rhizosphere soil samples. Each biological replicate originated from a separate sampling point, and the geographic information for individual sampling points is provided in Table S1. Sampling points within each soil category were located within a relatively small and environmentally comparable area. Rhizosphere soil collected from different sampling points was not pooled. Each biological replicate was processed independently for subsequent analyses. For soil physicochemical properties and enzyme activities, three technical measurements were performed for each biological replicate; the mean was used as the value of that biological replicate for statistical analysis. Technical replicates were not treated as independent observations. Statistical analyses were conducted using the six independently collected biological replicates within each soil category.

Each soil sample was divided into two portions. The first portion was used for analyzing soil physicochemical properties. Fresh rhizosphere soil was air-dried naturally, ground, and passed through a 30–50 mesh sieve as the first part of the sample. The second portion was stored at −80 °C for subsequent DNA extraction and microbial community analysis. In addition, the early stage of root rot was defined based on visible symptoms, including slight root discoloration and mild tissue rotting, without extensive root decay or above-ground wilting.

### 2.2. Soil Physicochemical Properties and Enzyme Activities

Soil available nitrogen (AN) was measured using the alkali hydrolysis diffusion method. Available phosphorus (AP) was determined by molybdenum–antimony anti-colorimetry, and available potassium (AK) was analyzed by flame atomic absorption spectrophotometry. Soil organic matter (OM) was quantified via the potassium dichromate external heating method, pH was measured with a pH meter, and total carbon (TC) was determined using a TOC analyzer. These parameters reflect fundamental soil characteristics [25,26].

Soil extracellular enzyme activities were determined using commercial assay kits (Solarbio Science & Technology Co., Ltd., Beijing, China), including sucrase (BC0245), amylase (BC1925), β-xylosidase (BC4015), β-glucosidase (BC0165), cellulase (BC0155), and laccase (BC1965). Soil samples were air-dried, ground, and sieved, with the assay samples prepared according to the manufacturer’s instructions. All samples were subjected to identical pretreatment conditions before analysis. All assays were conducted using six biological replicates. Briefly, 30 mg of soil was used for the sucrase assay and incubated at 37 °C for 24 h, with absorbance measured at 540 nm. Amylase activity was determined using 50 mg soil after incubation at 37 °C for 24 h and was measured at 540 nm. β-Xylosidase and β-glucosidase activities were determined using 20 mg of soil after incubation at 50 °C for 1 h and 37 °C for 1 h, respectively, with absorbance measured at 400 nm. Cellulase activity was determined using 50 mg of soil after incubation at 40 °C for 24 h and measured at 505 nm, whereas laccase activity was determined using 30 mg of soil after a 10 min reaction at 37 °C and measured at 420 nm. Enzyme activities were calculated from the corresponding absorbance values or standard curves according to the manufacturer’s protocols. Absorbance was measured using a Multiskan FC microplate reader (Thermo Fisher Scientific, Waltham, MA, USA).

Enzyme activities were calculated according to the following equations:

(i) Soil sucrase (S-SC):

$$S-SC\;activity\;\left(U/g\;soil\right)=x\times Vreaction\;/\left(W\times T\right)$$

where *x* is the glucose concentration calculated from the standard curve (mg mL^−1^), *Vreaction* is the total reaction volume (mL), *W* is the dry soil weight (g), and *T* is the reaction time (d).

(ii) Soil amylase (S-AL):

$$S-AL\;activity\;\left(U/g\;soil\right)=0.21\times x/W$$

where *x* is the concentration of reducing sugars calculated from the standard curve (μmol mL^−1^), and *W* is the dry soil weight (g).

(iii) Soil β-xylosidase (S-β-XYS):

$$S-\beta -XYS\;activity\;\left(U/g\;soil\right)=0.444\times \frac{\Delta Ameasured}{\Delta Astandard\times W}$$

where *ΔAmeasured* is the absorbance difference between the measured and control groups, *ΔAstandard* is the absorbance difference between the standard and blank groups, and *W* is the dry soil weight (g).

(iv) Soil β-glucosidase (S-β-GC):

$$S-\beta -GC\;activity\;\left(U/g\;soil\right)=0.72\times \Delta Ameasured\;/\left(\Delta Astandard\times W\right)$$

where *ΔAmeasured* is the absorbance difference between the measured and control groups, *ΔAstandard* is the absorbance difference between the standard and blank groups, and *W* is the dry soil weight (g).

(v) Soil cellulase (S-CL):

$$S-CL\;activity\;\left(U/g\;soil\right)=144\times x$$

where *x* is the glucose concentration calculated from the standard curve (mg mL^−1^).

(vi) Soil laccase (SL):

$$SL\;activity\;\left(U/g\;soil\right)=1.39\times \frac{\Delta Ameasured}{W}$$

where *ΔAmeasured* is the absorbance difference between the measured and control groups, and *W* is the dry soil weight (g).

The selected enzymes are key indicators of soil carbon cycling and organic matter decomposition processes, which are closely associated with rhizosphere microbial activity and plant–soil interactions.

### 2.3. DNA Extraction, PCR Amplification, and Sequencing

The total microbial genomic DNA was extracted from the samples of HLS, RRLS, and HCS using the TIANamp Soil DNA Kit (Tiangen Biotech, Beijing, China) according to the manufacturer’s instructions. DNA quality and concentration were assessed by 1.0% agarose gel electrophoresis and a NanoDrop2000 spectrophotometer (Thermo Scientific, Waltham, MA, USA). Extracted DNA was stored at −80 °C until further use. For microbial community analysis, six independent biological replicates were analyzed for each soil category (HLS, RRLS, and HCS), resulting in a total of 18 rhizosphere soil samples for DNA extraction and amplicon sequencing. Each biological replicate was processed independently throughout DNA extraction, PCR amplification, and sequencing.

The hypervariable V3-V4 region of the bacterial 16S rRNA gene was amplified with primers 341F (5′-CCTAYGGGRBGCASCAG-3′) and 806R (5′-GGACTACHV GGGTWTCTAAT-3′) [27]. The ITS1-5F region of the fungal ITS rRNA gene was amplified with primers 1737F (5′-GGAAGTAAAAGTCGTAACAAGG-3′) and 2043R (5′-GCTGCGTTCTTCATCGATGC-3′) using a T100 Thermal Cycler (BIO-RAD, California, USA). The PCR mixture contained 4 μL of 5× Fast Pfu buffer, 2 μL of 2.5 mM dNTPs, 0.8 μL of each primer (5 μM), 0.4 μL of Fast Pfu polymerase, 10 ng of template DNA, and ddH_2_O to a final volume of 20 μL. The PCR conditions were as follows: initial denaturation at 95 °C for 3 min; 27 cycles of denaturation at 95 °C for 30 s, annealing at 55 °C for 30 s, and extension at 72 °C for 45 s; final extension at 72 °C for 10 min; and holding at 4 °C. PCR products were separated on a 2% agarose gel, purified using the PCR Clean-Up Kit (YuHua, Shanghai, China), and quantified with Qubit 4.0 (Thermo Fisher Scientific, Waltham, MA, USA).

Purified amplicons were pooled in equimolar concentrations and subjected to paired-end sequencing on an Illumina NextSeq2000 platform (Illumina, San Diego, CA, USA) by Majorbio Bio-Pharm Technology Co., Ltd. (Shanghai, China), following standard protocols. Raw sequencing reads have been submitted to the NCBI Sequence Read Archive (SRA) (BioProject ID: PRJNA1353648).

### 2.4. Data Processing and Statistical Analysis

For physicochemical and enzyme-activity variables, the mean of three technical measurements was calculated for each biological replicate before statistical analysis. Thus, *n* = 6 independent biological replicates per soil category. Data were assessed for normality using the Shapiro–Wilk test and for homogeneity of variance using Levene’s test. Variables satisfying these assumptions were analyzed using one-way ANOVA followed by Tukey’s HSD test.

Raw sequences were quality-filtered using fastp v0.19.6 and merged using FLASH v1.2.11 [28,29]. Reads were truncated when the mean quality score within a 50-bp sliding window fell below 20. Reads shorter than 50 bp after trimming or containing ambiguous bases were discarded. Paired-end reads with an overlap longer than 10 bp and a maximum mismatch ratio of 0.2 were merged; unmerged reads were discarded. Samples were demultiplexed according to their barcodes and primer sequences, allowing up to two nucleotide mismatches in primer matching.

Operational taxonomic units (OTUs) were clustered using UPARSE v. 11 at 97% similarity, and chimeric sequences were removed [30]. To mitigate the influence of sequencing depth on alpha- and beta-diversity analyses, all samples were rarefied to 20,000 sequences per sample, which retained over 97% average sequence coverage. Sequencing depth ranged from 84,121 to 100,762 high-quality reads per sample, with a mean of 93,375 ± 4200 reads (mean ± SD). Rarefaction curves based on the Sobs index at the genus level showed a progressively decreasing rate of increase with increasing sequencing depth, indicating sufficient sequencing effort. Rarefaction curves are provided in Figures S3 and S4. Taxonomic annotation of OTUs was performed using the RDP classifier against the SILVA 16S rRNA database (v. 138) for bacteria and the UNITE database (v. 8.0) for fungi, with a confidence threshold of 70%. Alpha-diversity indices, including Chao1, Simpson, and Shannon, were calculated using Mothur v. 1.30.2 [31].

Beta-diversity patterns were visualized using principal coordinates analysis (PCoA) based on Bray–Curtis dissimilarity with the vegan package (v2.5-3) in R. Differences in bacterial and fungal community composition among soil categories were further evaluated using analysis of similarities (ANOSIM) based on Bray–Curtis dissimilarity. Statistical significance was assessed using permutation testing (9999 permutations). Distance-based redundancy analysis (dbRDA) was conducted with the capscale function in the vegan package (v2.6-4) for R [32]. Functional annotation of fungal communities was performed using FUNGuild v1.0, and bacterial functional profiles were predicted using PICRUSt2 v2.2.0. Briefly, 16S rRNA gene copy numbers were first predicted, and OTU abundances were normalized according to the predicted 16S rRNA gene copy numbers before metagenome prediction. OTUs with a nearest-sequenced taxon index (NSTI) exceeding the default maximum threshold of 2.0 were excluded from downstream functional prediction. Predicted pathway abundances were subsequently generated from the inferred metagenomic profiles. Differences in predicted pathway abundance among the three soil groups were evaluated using the Kruskal–Wallis test, and *p*-values were adjusted for multiple comparisons using the Benjamini–Hochberg (BH) procedure to control the false discovery rate (FDR) [33,34]. All physiological and biochemical data were analyzed using one-way analysis of variance followed by Tukey’s honestly significant difference (HSD) test at *p* < 0.05. Where multiple hypotheses were tested simultaneously, *p*-values were again adjusted using the Benjamini–Hochberg FDR procedure, with adjusted *p* < 0.05 considered statistically significant.

For the fungal and bacterial dbRDA, 18 independent biological replicates were analyzed (*n* = 6 per soil category). The constrained ordination included pH, available nitrogen (AN), available phosphorus (AP), available potassium (AK), organic matter (OM), total carbon (TC), and the activities of saccharase, amylase, β-xylosidase, β-glucosidase, cellulase, and laccase. Environmental vectors were fitted to the ordination, and the 12 resulting *p*-values were adjusted using the Benjamini–Hochberg FDR procedure. Because the supplied analysis output did not contain variance-inflation factors or a documented variable-selection procedure, the dbRDA was interpreted as a multivariate association analysis rather than evidence for independent or causal environmental effects. Spearman correlations between the 10 dominant taxa at each taxonomic level and the 12 measured soil variables were initially calculated across the 18 samples. To evaluate whether these associations were attributable to differences among soil categories, taxon abundances and environmental variables were rank-transformed and residualized against indicator variables for HLS, RRLS, and HCS; correlations between the resulting residuals were then calculated as group-adjusted partial Spearman correlations. Statistical significance was evaluated using 9999 permutations restricted within each soil category. For both the pooled and group-adjusted analyses, *p*-values were adjusted jointly across the 480 taxon-environment comparisons using the Benjamini–Hochberg FDR procedure, with adjusted *p* < 0.05 considered statistically significant.

## 3. Results

### 3.1. Values of pH, Available Nitrogen, Phosphorus, Potassium, and Organic Matter in Different Soil Habitats

**Figure 1:**
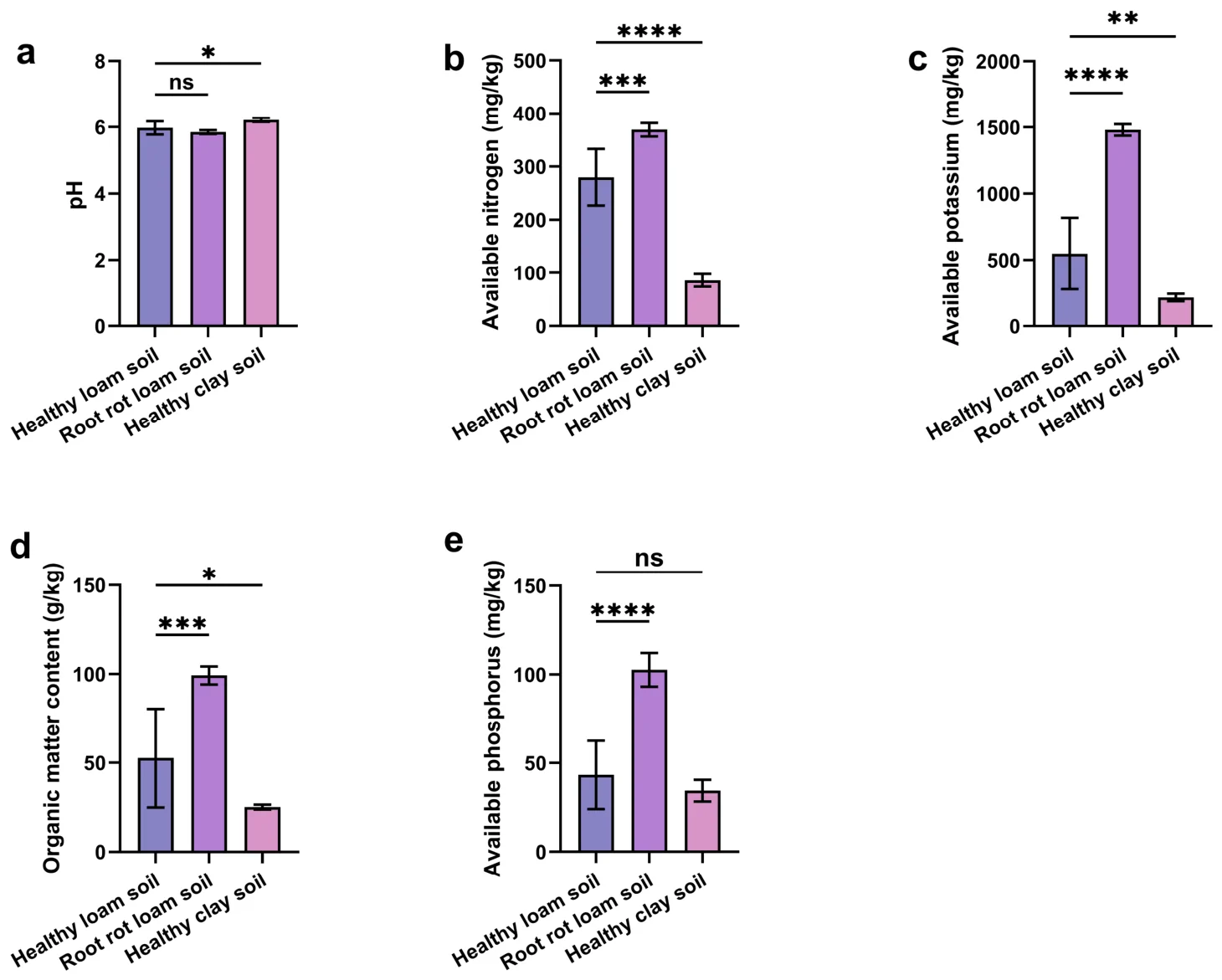
Physicochemical properties of the three soil samples. (**a**) pH; (**b**) available nitrogen; (**c**) available potassium; (**d**) organic matter content; (**e**) available phosphorus. Data are presented as mean ± SD (*n* = 6 independent biological replicates per group). (ns: no significant difference; * *p* < 0.05; ** *p* < 0.01; *** *p* < 0.001; **** *p* < 0.0001).

The pH, available nitrogen, phosphorus, potassium, and organic matter were compared among the three soil habitats. Results demonstrated that there was no significant difference in the pH values of the three soil habitats (mean pH, HLS: 5.975, RRLS: 5.852, HCS: 6.22) (Figure 1a). However, there were significant differences between HLS and RRLS (*p* < 0.01) and HLS and HCS (*p* < 0.0001) when comparing available nitrogen (mean available nitrogen content (mg/kg), HLS: 279.949, RRLS: 369.184, HCS: 85.704) (Figure 1b); between HLS and RRLS (*p* < 0.0001) and HLS and HCS (*p* < 0.05) when comparing available potassium (average available potassium content (mg/kg), HLS: 548.750, RRLS: 1481.294, HCS: 216.156) (Figure 1c); and between HLS and RRLS (*p* < 0.05), and HLS and HCS (*p* < 0.01) when comparing organic matter (average organic matter (g/kg), HLS: 52.679, RRLS: 99.296, HCS: 25.159) (Figure 1d) (Table S2). As for available phosphorus, significant differences were found between HLS and RRLS (*p* < 0.001) but not between HLS and HCS (mean available phosphorus content (mg/kg), HLS: 43.305, RRLS: 102.695, HCS: 34.424) (Figure 1e).

### 3.2. Bacterial Communities on Rhizosphere of A. lappa in Different Soil Environments

In this study, we observed 7198 bacterial OTUs and 5314 fungal OTUs in HLS; 8068 bacterial OTUs and 3086 fungal OTUs in RRLS; 9431 bacterial OTUs and 1616 fungal OTUs in HCS. This gave a total of 15,905 bacterial OTUs and 7026 fungal OTUs observed across the three groups (Tables S3 and S4, Figure S1).

Bacterial α-diversity was relatively stable among the three soil habitats, although bacterial richness, as indicated by the Chao1 index, was significantly higher in RRLS than in HLS (*p* < 0.05). No significant differences were observed in bacterial Simpson or Shannon indices. Fungal α-diversity did not differ significantly between RRLS and HLS, whereas HCS exhibited significantly lower Chao1 and Shannon indices and a higher Simpson index than HLS (Table 1 and Table S5).

**Figure 2:**
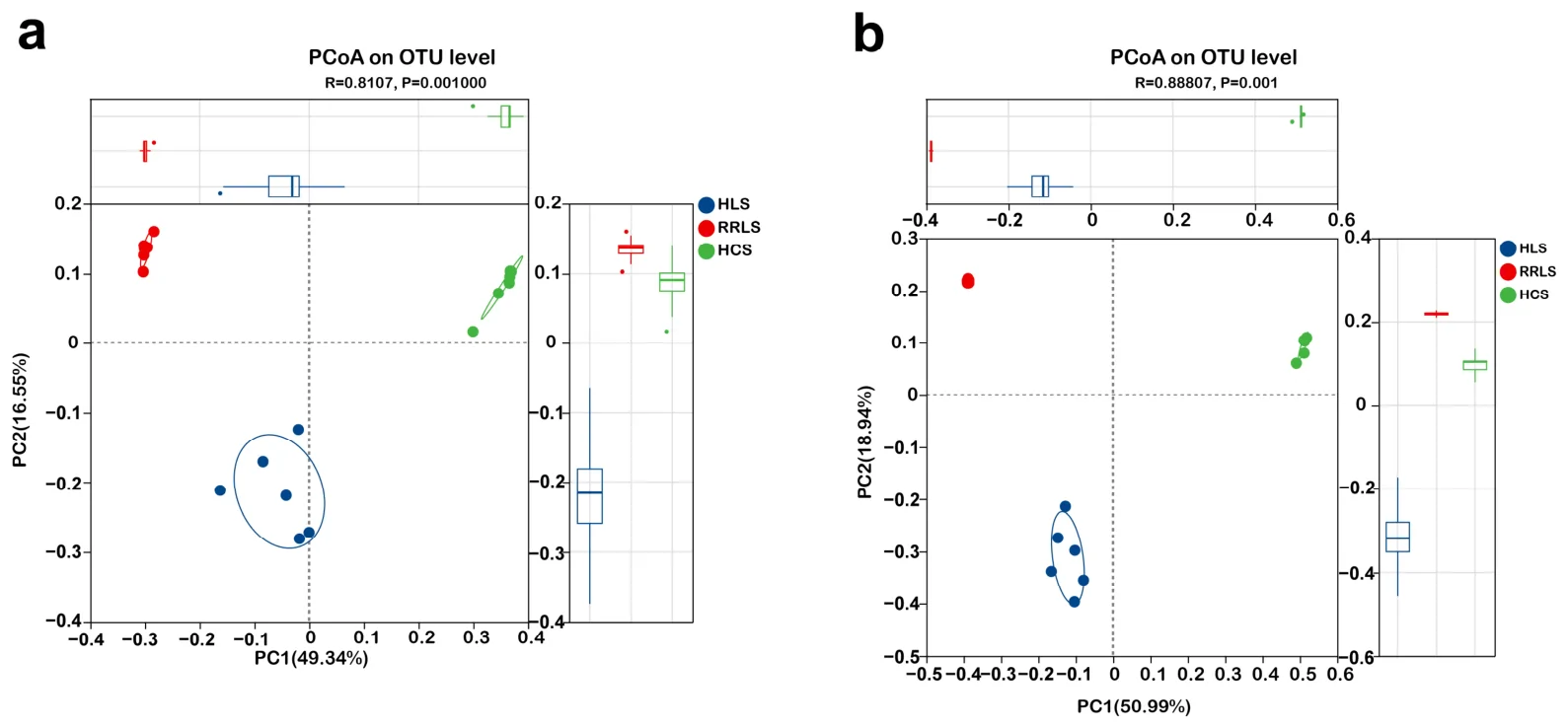
Distribution of bacterial and fungal communities in three groups (principal coordinate analysis). (**a**) Bacterial community in the rhizosphere soil of *Aucklandia lappa*; (**b**) fungal community in the rhizosphere soil of *A. lappa.* Each point represents one biological replicate (*n* = 6 per group).

The distributions of bacterial and fungal communities are shown in Figure 2. The horizontal axis accounts for 49.34% (Figure 2a) and 50.99% (Figure 2b) of bacterial and fungal communities, respectively, while the vertical axis only explains 16.55% and 18.94% of bacterial and fungal communities, respectively. The three habitats formed relatively independent distribution areas, and ANOSIM further confirmed significant differences among the three habitats for bacteria (r = 0.8107, *p* = 0.001) and fungi (*r* = 0.8881, *p* = 0.001). PCoA based on Bray–Curtis dissimilarity showed clear separation among the three soil habitats for both bacterial and fungal communities (Figure 2). The first two axes explained 65.89% of the variation in bacterial community composition and 69.93% of the variation in fungal community composition. Statistical analysis further indicated significant differences in community composition among the three habitats (*p* = 0.001).

**Figure 3:**
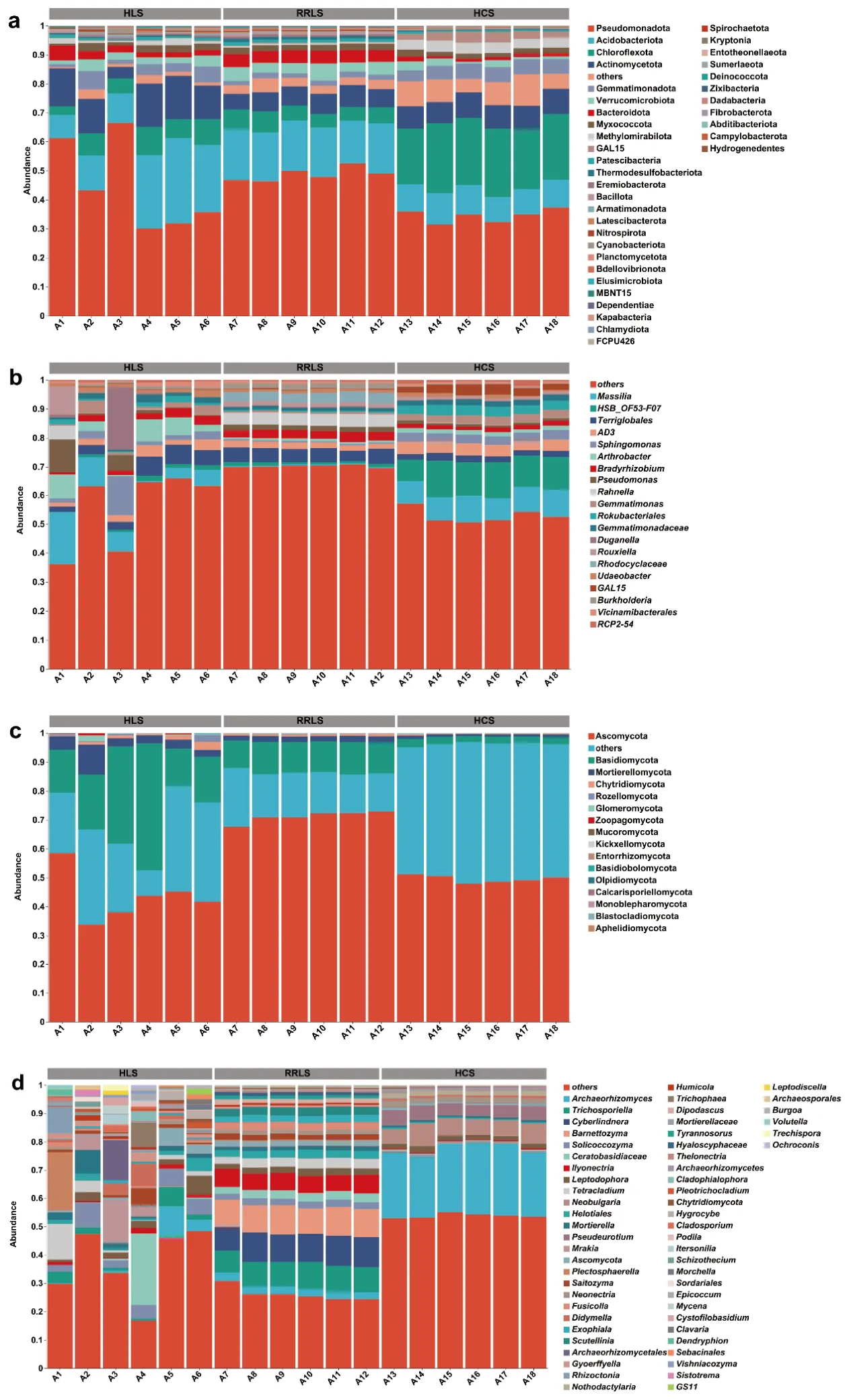
Analysis of microbial community composition in each soil sample at phylum and genus levels: (**a**) bacterial phylum level; (**b**) bacterial genus level; (**c**) fungal phylum level; and (**d**) fungal genus level.

**Figure 4:**
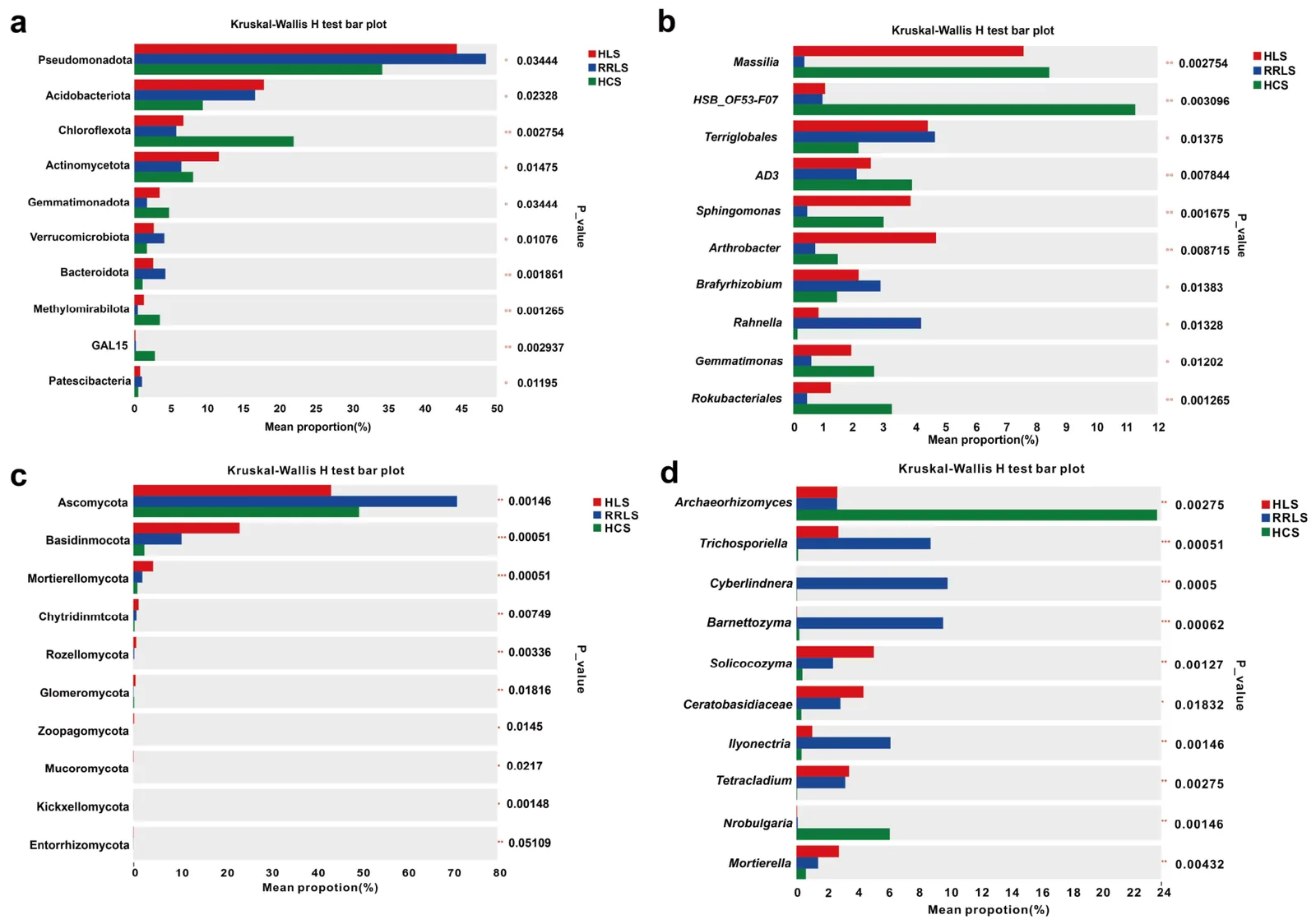
The top 30 operational taxonomic units were compared with SILVA and UNITE databases to analyze dominant bacteria and fungi in different groups at phylum and genus levels: (**a**) dominant bacterial phyla; (**b**) dominant bacterial genera; (**c**) dominant fungal phyla; (**d**) dominant fungal genera.

The dominant bacteria in HLS and RRLS were the same at the phylum level but differed in proportion. For example, the relative abundance of Pseudomonadota was 29.88–66.20% in HLS and 46.15–52.14% in RRLS. The relative abundance of Acidobacteriota was 8.06–29.12% in HLS and 14.60–17.36% in RRLS. The relative abundance of Actinomycetota was 3.88–14.92% in HLS and 5.35–7.49% in RRLS. Slightly different from those in the above two habitats, the dominant bacteria in HCS were Pseudomonadota (relative abundance 31.25–37.36%), Chloroflexota (relative abundance 18.83–23.91%), and Acidobacteriota (relative abundance 8.47–10.70%) (Figure 3a and Figure 4a, Table S6).

At the genus level, the dominant bacterial genera in HLS were *Massilia*, *Arthrobacter*, and *Terriglobales*. The dominant bacterial genera in RRLS were *Terriglobales*, *Rahnella,* and *Rhodocyclaceae*, while the dominant bacterial genus in HCS was *Massilia* along with several unclassified bacteria (Figure 3b and Figure 4b, Table S7).

### 3.3. Fungal Communities in Rhizosphere of A. lappa in Different Soil Environments

The dominant fungi in HLS, RRLS, and HCS were the same at the phylum level but different in proportion. For example, the proportion of Ascomycota in HLS, RRLS, and HCS was 33.83–58.53%, 67.54–72.87%, and 47.91–51.24%, respectively; the proportion of Basidiomycota in HLS, RRLS, and HCS was 12.88–43.87%, 9.52–11.23%, and 2.05–2.83%, respectively; and the proportion of Mortierellomycota in HLS, RRLS, and HCS was 2.37–10.39%, 1.64–2.19%, and 0.70–1.11%, respectively (Figure 3c and Figure 4c, Table S8).

At the genus level, the dominant fungi in HLS were *Solicoccozyma* and *Mrakia*; in RRLS, they were *Cyberlindnera*, *Barnettozyma,* and *Trichosporiella*; and in HCS, they were *Archaeorhizomyces*, *Neobulgaria*, and *Pseudeurotium* (Figure 3d and Figure 4d, Table S9).

### 3.4. Enzymatic Activities and Carbon Accumulation in Different Soil Habitats

An analysis of soil enzyme activities revealed that, compared with HLS, the activities of soil cellulase (SCL) and soil saccharase (SSC) were significantly increased in RRLS (increased by 54.83%, *p* < 0.05 for SCL; increased by 295.13%, *p* < 0.0001 for SSC), while significantly decreased in HCS (decreased by 78.23%, *p* < 0.01 for SCL; decreased by 58.82%, *p* < 0.001 for SSC).

**Figure 5:**
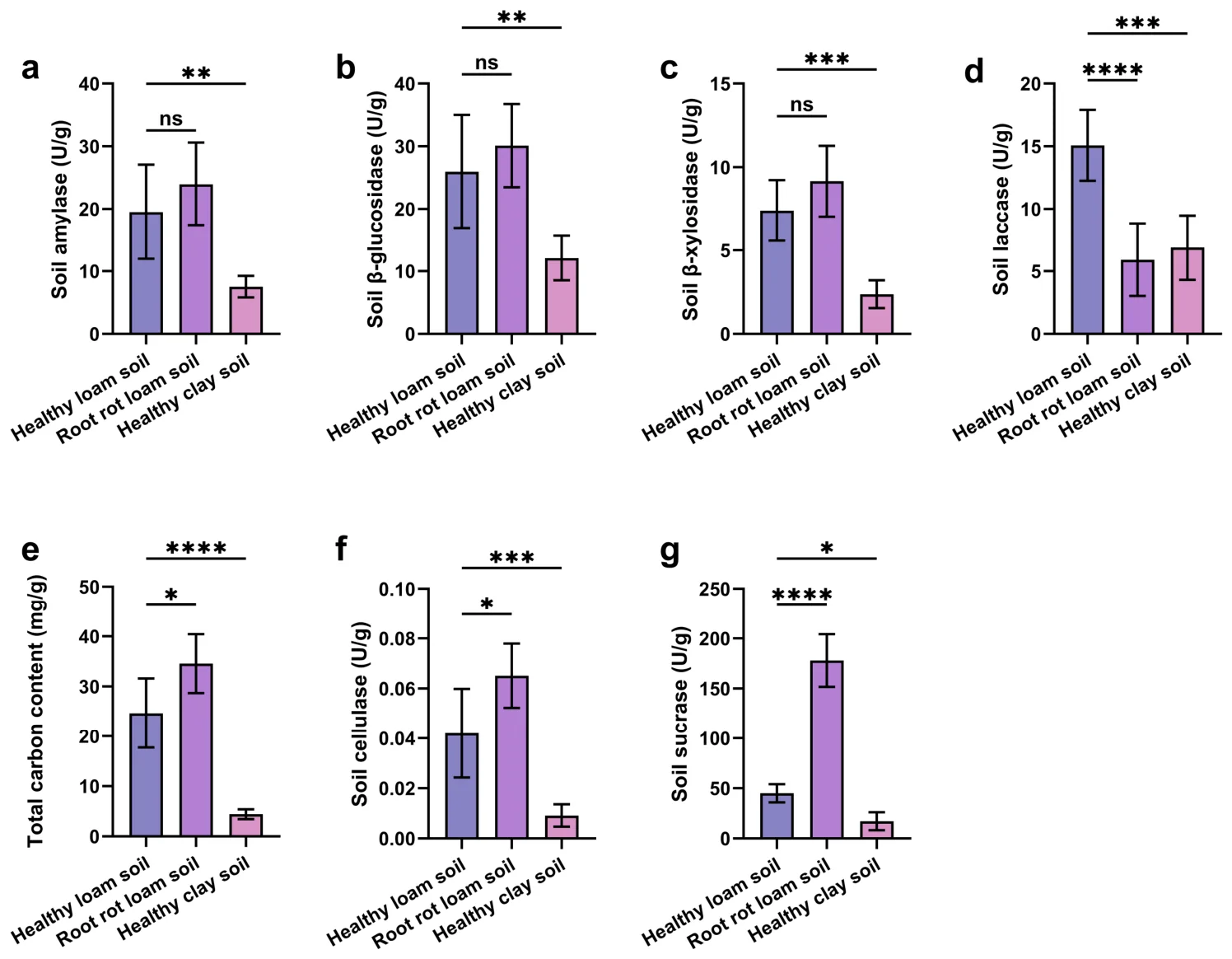
Enzyme activity and total carbon content of three soil samples (HLS, RRLS, and HCS). (**a**) Soil amylase; (**b**) soil-β-glucosidase; (**c**) soil-β-xylosidase; (**d**) soil laccase; (**e**) total carbon; (**f**) soil cellulase; (**g**) soil saccharase. Data are presented as mean ± SD (*n* = 6 independent biological replicates). (ns: no significant difference; * *p* < 0.05; ** *p* < 0.01; *** *p* < 0.001; **** *p* < 0.0001).

The activity trends of soil amylase (SAL), soil-β-Glucosidase (S-β-GC) and soil-β-xylosidase (S-β-XYS) were similar in HLS, RRLS, and HCS, i.e., the activities of SAL, S-β-GC, and S-β-XYS were not significantly different between HLS and RRLS, but their activities were all significantly decreased in HCS in comparison with HLS (decreased by 61.44%, *p* < 0.01 for SAL; decreased by 53.42%, *p* < 0.01 for S-β-GC; decreased by 67.96%, *p* < 0.01 for S-β-XYS). It is noteworthy that, compared with HLS, soil laccase (SL) activity decreased significantly in RRLS (by 62.04%, *p* < 0.001) and HCS (by 55.74%, *p* < 0.001) (Figure 5a–d); moreover, the value of total carbon was significantly different between HLS and RRLS (*p* < 0.05) and HLS and HCS (*p* < 0.0001), with average values of 24.67 mg/g in HLS, 34.56 mg/g in RRLS, and 4.43 mg/g in HCS (Figure 5e) (Table S10).

By comparison, the total carbon value in RRLS was higher than that in HLS, while the total carbon value in HCS was lower than that in HLS. With the increase in total carbon, the enzyme activities of SAL, SCL, SSC, S-β-GC, and S-β-XYS all significantly increased (Figure 5a–c,f–g). Compared with RRLS, the total carbon content decreased in HCS, which was accompanied by a decrease in the enzyme activities of SAL, SCL, SSC, S-β-GC, and S-β-XYS, but the enzyme activity of SL increased.

### 3.5. Correlation Between Soil Enzyme Activity, Physicochemical Properties, and Dominant Bacteria in Different Soil Habitats

**Figure 6:**
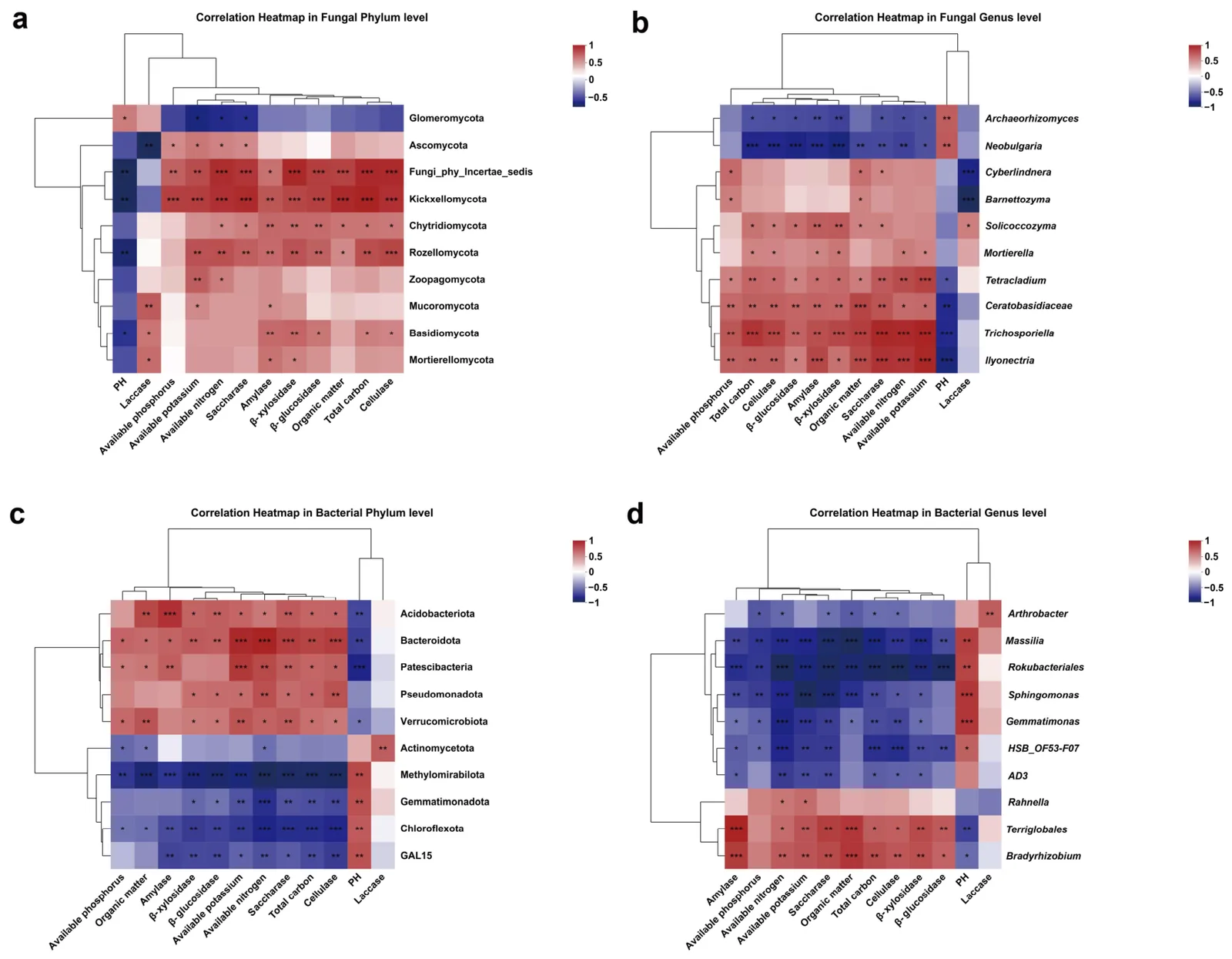
Spearman correlation test between microorganisms and various parameters. (**a**) Fungal phylum level; (**b**) fungal genus level; (**c**) bacterial phylum level; (**d**) bacterial genus level.

A Spearman test was performed to understand the coupling relationship among physicochemical properties, enzyme activity, and the dominant microorganism. The results demonstrated that Glomeromycota was significantly and negatively correlated with all the parameters, excluding pH and laccase, at the fungal phylum level, while Ascomycota and Kickxellomycota were exactly the opposite of Glomeromycota and had a positive relationship with all parameters except pH and laccase; all remaining dominant phyla had a positive relationship with the parameters, excluding pH. At the fungal genus level, *Archaeorhizomyces* and *Neobulgaria* were positively correlated with pH and negatively correlated with all the other remaining elements (available phosphorus, available nitrogen, available potassium, total carbon, cellulase, glucosidase, amylase, xylosidase, organic matter, saccharase, and laccase), while *Solicoccozyma*, *Mortierella,* and *Helotiaceae* were negatively correlated with pH and positively correlated with all other elements; all remaining dominant genera were negatively correlated with pH and laccase and positively correlated with all other elements (Figure 6a,b).

As for bacteria, Acidobacteriota was negatively correlated with pH but positively correlated with all other elements at the bacterial phylum level; Bacteroidota, Patescibacteria, Pseudomonadota, and Verrucomicrobiota were negatively correlated with pH and laccase but positively correlated with all other elements. However, the correlations of Actinomycetota, Methylomirabilota, and Gemmatimonadota with all elements were completely opposite to the correlations of the four bacterial phyla mentioned above with all elements. In addition, Chloroflexota and GAL15 were positively correlated with pH and negatively with all other elements. At the bacterial genus level, *Arthrobacter*, *Massilia*, *Terriglobales*, *Sphingomonas*, and *Gemmatimonas* were positively correlated with pH and laccase but negatively correlated with all other elements; *Rahnella* and *Bradyrhizobium* were negatively correlated with pH and laccase and positively correlated with all other elements, while *Terriglobales* was negatively correlated with pH but positively correlated with all other elements (Figure 6c,d). Across the four pooled correlation matrices, 326 of 480 comparisons had unadjusted *p*-values < 0.05, and 284 remained significant after joint Benjamini–Hochberg correction. However, adjustment for soil category substantially attenuated the correlations: the median absolute correlation coefficient decreased from 0.486 to 0.192 for fungal phyla, from 0.571 to 0.260 for fungal genera, from 0.577 to 0.213 for bacterial phyla, and from 0.598 to 0.201 for bacterial genera. Only 27 of the 480 group-adjusted associations had permutation *p*-values < 0.05 before correction, and none remained significant after Benjamini–Hochberg correction. Thus, the pooled heatmap patterns were largely structured by differences among HLS, RRLS, and HCS and should not be interpreted as within-category or mechanistic taxon–environment relationships.

**Figure 7:**
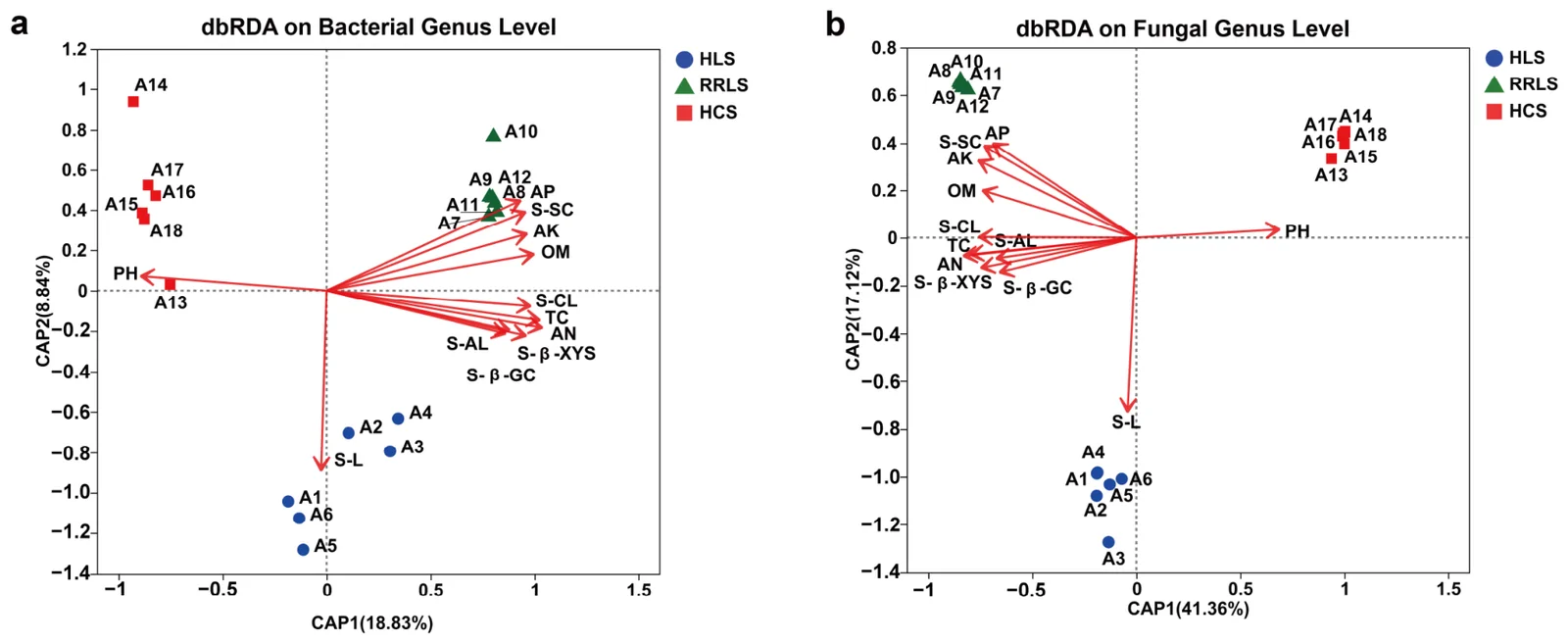
The relationship between soil bacteria (**a**) and fungi (**b**) and environmental variables.

The fungal dbRDA included 18 independent biological replicates (*n* = 6 per soil category) and 12 measured soil physicochemical and enzyme variables. The constrained component accounted for 89.895% of the total variation in fungal community composition. CAP1 and CAP2 explained 41.36% and 17.124% of the total variation, respectively, with a cumulative proportion of 58.484% (Figure 7a,b). Environmental vector fitting yielded R^2^ values ranging from 0.6065 to 0.9496, and all 12 fitted vectors remained significant after Benjamini–Hochberg correction (adjusted *p* = 0.0012–0.0040). Saccharase activity (R^2^ = 0.9496, adjusted *p* = 0.0012), AN (R^2^ = 0.9487, adjusted *p* = 0.0012), and AK (R^2^ = 0.9454, adjusted *p* = 0.0012) showed the highest fitted R^2^ values. These results indicate multivariate associations and do not establish independent or causal environmental effects.

### 3.6. Functional Interactions Among Microbial Communities, Soil Properties, and Enzyme Activities

**Figure 8:**
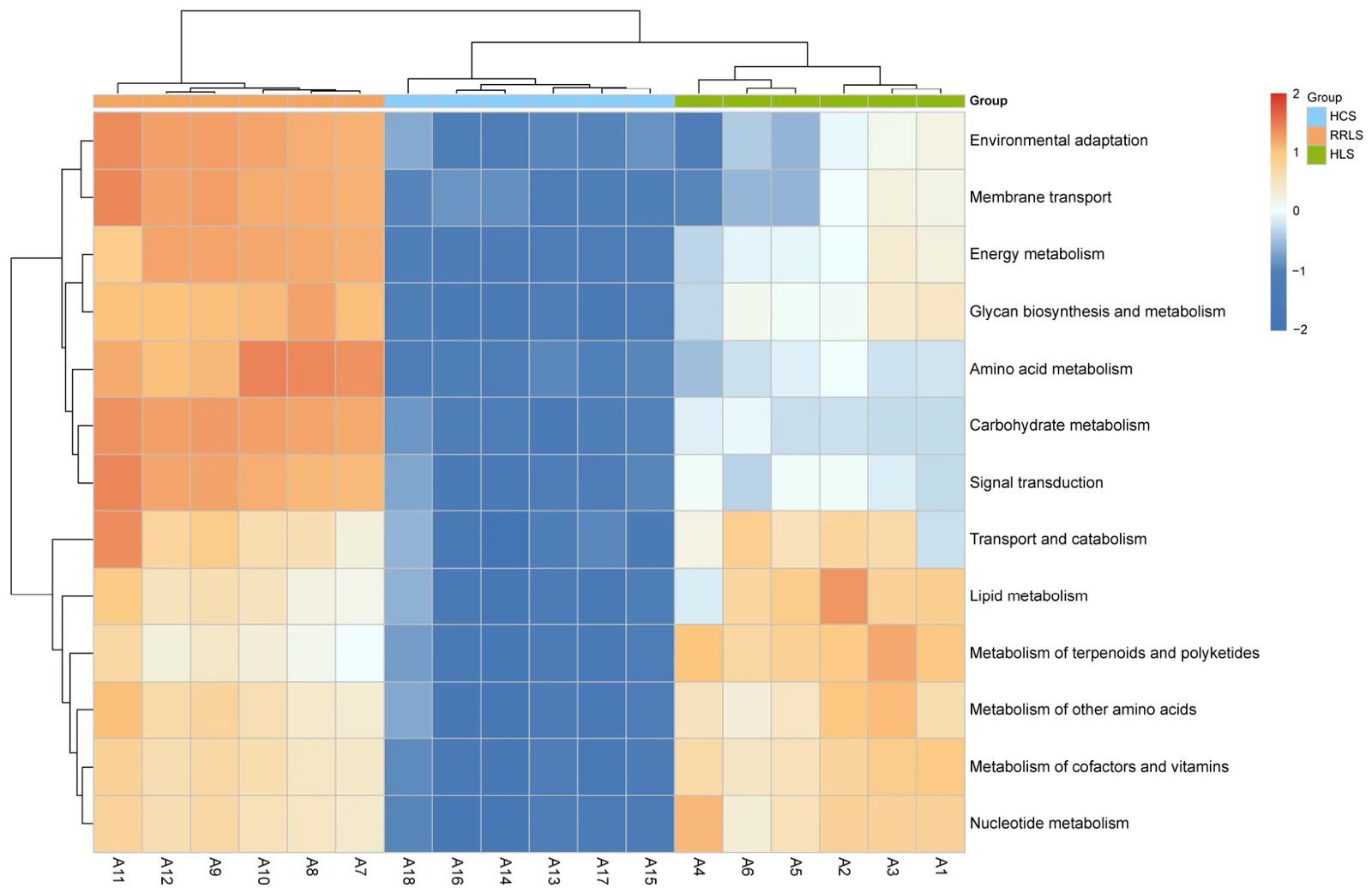
KEGG level 2 functional predictions of bacteria in different groups.

PICRUSt2 analysis indicated differences in predicted KEGG level 2 functional profiles among the three rhizosphere soil groups. Compared with HLS, RRLS showed higher predicted relative abundances of several functional categories, including membrane transport, energy metabolism, glycan biosynthesis and metabolism, amino acid metabolism, carbohydrate metabolism, and signal transduction, whereas lower predicted abundances of several of these categories were observed in HCS (Figure 8).

**Figure 9:**
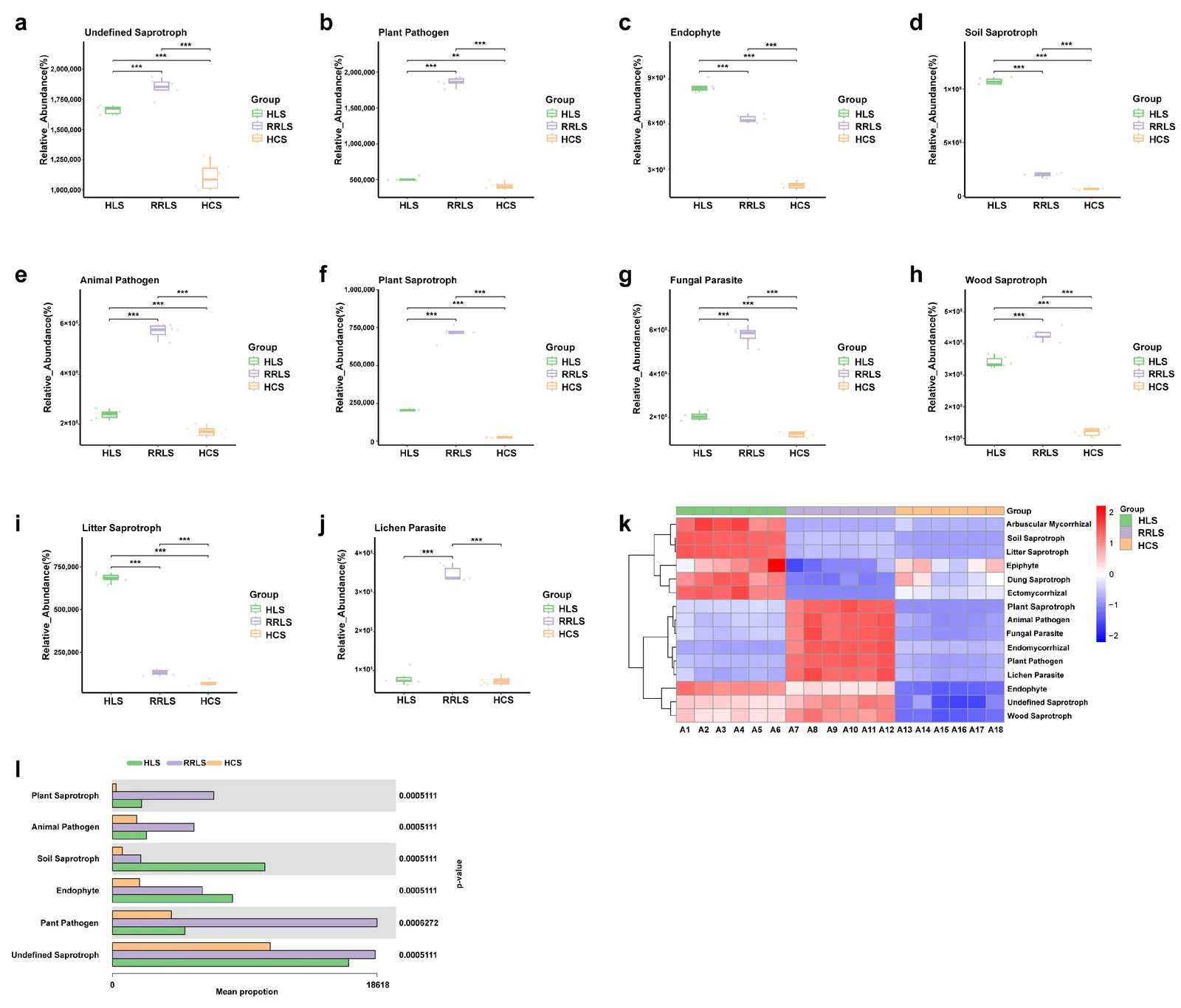
Functional prediction of fungal communities by FUNGuild. (**a**–**j**) Content of the top 10 different fungal functions in three soil types; (**k**) heatmap of the top 15 different fungal functions in three soil types; (**l**) absolute abundance of fungal functional guilds (FUNGuild annotation) across different treatments (HLS, RRLS, and HCS). Differences among treatments were evaluated using the Kruskal–Wallis test. A *p*-value of <0.05 was considered statistically significant.

A correlation analysis demonstrated strong coupling relationships between predicted microbial functions and soil physicochemical properties/enzyme activities. Carbohydrate metabolism, energy metabolism, amino acid metabolism, glycan biosynthesis and metabolism, membrane transport, and signal transduction were positively correlated with available nitrogen, potassium, organic matter, total carbon, cellulase, saccharase, β-glucosidase, and β-xylosidase, but negatively correlated with pH (Figure S2). In contrast, laccase activity showed generally negative correlations with several microbial functional categories. These results suggest that nutrient enrichment and enhanced carbon-cycle enzyme activities may be associated with rhizosphere microbial functional responses under root rot conditions. FUNGuild analysis indicated differences in the predicted ecological guild composition of fungal communities among the three rhizosphere soil groups (Figure 9). RRLS showed a higher relative representation of sequences assigned to plant-pathogen- and saprotroph-associated guilds, whereas HLS showed a higher relative representation of several symbiotrophic guilds, including arbuscular mycorrhizal fungi.

## 4. Discussion

### 4.1. Physicochemical Properties in Different Soil Habitats

In the present study, it was found that there were significant differences in key physicochemical properties among the three soil types (HLS, RRLS, and HCS). Specifically, HLS and RRLS had higher levels of available nitrogen, phosphorus, and potassium, as well as organic matter, while HCS had lower levels of available nitrogen and organic matter. We infer that these differences might be related to the higher nutrient levels observed in RRLS, which may be consistent with nutrient release associated with the degradation of damaged root tissues. However, because this study was cross-sectional, temporal precedence cannot be established, and pre-existing nutrient conditions may also have influenced plant health or disease susceptibility. As this study aimed to characterize rhizosphere factors associated with root rot status, all root and soil samples were collected at the early stage of root rot, at which stage the roots decay and decompose, and the nutrients increase; meanwhile, although some microbial decomposers may be suppressed during disease progression, the increased availability of easily degradable substrates from damaged root tissues may temporarily stimulate specific extracellular enzyme activities.

Fan et al. (2024) showed that the content of available potassium and other substances in *Atractylodes macrocephala* root-rot soil was higher than that in healthy soil, similar to our results [35]. The study by He et al. (2018) indicated that the levels of available potassium, available phosphorus and organic matter in the soil of *Amorphophallus konjac*, affected by rhizosphere soft rot, were higher than those in healthy soil, which is also consistent with our findings [36].

A previous study of Yang et al. (2023) revealed that the content of organic matter and total carbon in *Psammosilene tunicoides* rhizosphere soil with root rot was higher than those of healthy plant soil, but the pH, available phosphorus, and available potassium content were reduced, which is partly consistent with our current results [37]. Liao et al. (2022) showed that prickly ash root-rot soil has lower total nitrogen and available phosphorus content and increased pH compared to normal soil, which is different from our results [38]. The study by Wang et al. (2025) on the root rot of *Annona squamosa* revealed that the pH and organic matter contents in the soil affected by root rot were significantly lower than those of healthy soil, while the content of available nitrogen increased [39]. The changes they observed in pH and available nitrogen were consistent with our results, though the changes in organic matter content were opposite to our findings. It is speculated that the soil physical and chemical properties with root rot plants may vary depending on the plants.

Interestingly, some of our findings differed from previous studies reporting decreased nutrient availability and enzyme activities under root rot or soil-borne disease conditions. Several factors may explain these discrepancies. First, the present study focused on the early stage of root rot, during which root tissue degradation may temporarily release nutrients into the rhizosphere, potentially resulting in short-term nutrient enrichment and increased enzyme activities. In contrast, many previous studies investigated later disease stages, characterized by severe root deterioration, nutrient depletion, and the disruption of soil microbial functions. Second, differences in soil type, host plant species, and environmental conditions may also contribute to inconsistent ecological responses. Notably, this study was conducted in mountainous habitats and focused on rhizosphere soils, which are strongly influenced by root activity and localized microbial processes, whereas many previous studies were based on bulk soil systems. Thirdly, variations in microbial community composition and metabolic responses may further contribute to different nutrient and enzyme activity patterns under similar disease pressures. Therefore, the discrepancies observed in this study are more likely to reflect context-dependent rhizosphere responses during early root rot rather than direct contradictions to previous findings, highlighting the importance of considering disease stage when interpreting soil ecological responses to root rot.

Although clay soils are generally considered to possess stronger nutrient-retention capacity than loam soils, our results showed relatively lower levels of available nutrients in healthy clay rhizosphere soils. This apparent inconsistency may arise because nutrient-retention capacity does not necessarily correspond to the measured pool of bioavailable nutrients. In field rhizosphere systems, nutrient availability is influenced by multiple interacting factors, including plant nutrient uptake, root exudation, microbial mineralization, organic matter turnover, fertilization history, and nutrient leaching. Previous studies have shown that the nutrient levels in rhizosphere soils may differ substantially from those in bulk soils. For example, Cui et al. (2021) reported relatively lower nutrient concentrations in rhizosphere soils compared with surrounding bulk soils in alpine ecosystems, suggesting that root-associated processes may substantially modify local nutrient availability [40]. In the present study, the compact structure and lower aeration typically associated with clay soils may have reduced nutrient turnover and microbial mineralization, potentially contributing to lower levels of available N, K, organic matter, and total carbon found in the healthy clay soils compared with the healthy loam soils. Nevertheless, because detailed information regarding fertilization history and soil parent material was unavailable, the comparison between HLS and HCS should be interpreted cautiously as a field-level rhizosphere difference rather than evidence that clay soils generally contain lower nutrient levels than loam soils.

### 4.2. Diversity of Microorganisms in Different Soil Habitats

Sequencing analysis in our current study found that the diversity and richness of microorganisms in healthy loam soil were comparable to those in root rot loam soil, but the diversity and richness of microorganisms in healthy clay soil were significantly reduced. Although several previous studies have shown that soil microbial diversity in a healthy plant habitat is always higher than that in a diseased plant habitat, such as *Aconitum carmichaeli* [41], *Panax notoginseng* [42], and *P. ginseng* [43], our results revealed that the soil microbial environments of healthy and diseased plants grown in loam were similar in diversity and abundance, which was consistent with the results of Yang et al. (2023) [37].

Overall, bacterial diversity was relatively similar between HLS and RRLS, although the bacterial richness estimated by the Chao1 index was modestly but significantly higher in RRLS. In contrast, fungal α-diversity did not differ significantly between these two loam-soil groups. This may be attributed to the early stage of root rot infection, where native soil microorganisms are inhibited in activity but still present in the diseased area, while pathogenic bacteria have emerged but not yet spread extensively, thus limiting the survival of normal microbes. In contrast, the decrease in diversity and richness observed in clay soil may be due to poor air permeability and low nutrient content, which are unfavorable for both plant and microbial survival.

At the genus level, *Rahnella* was relatively more abundant in RRLS than in HLS. Members of *Rahnella* are widely distributed in soil and plant-associated environments, and some strains have been reported to exhibit plant growth-promoting or antagonistic activities against phytopathogens [44,45]. Therefore, the enrichment of *Rahnella* in RRLS does not necessarily indicate a pathogenic role and may instead represent a microbial response to altered rhizosphere conditions or plant disease.

Several fungal genera, including *Trichosporiella*, *Cyberlindnera*, *Barnettozyma*, and *Ilyonectria*, were also relatively enriched in RRLS. Among them, species of *Ilyonectria* have previously been associated with root diseases of several medicinal plants, including *P. quinquefolius* and *P. ginseng* [46,47,48]. Nevertheless, the enrichment of *Ilyonectria* sequences in the present study cannot by itself demonstrate that this genus causes root rot in *A. lappa*. Establishing pathogenicity would require isolation of representative strains, followed by controlled inoculation and pathogenicity testing. Conversely, some members of *Cyberlindnera* and related yeast taxa have been reported to exhibit antagonistic activities against plant pathogens. Thus, the taxa enriched in RRLS may include organisms associated with disease development, decomposition of damaged root tissues, or microbial responses to altered rhizosphere conditions. Their specific ecological roles in the *A. lappa* root rot system require further experimental validation.

Similarly, Chiu et al. (2018) found that polysaccharides produced by *Trichosporon* sp. can antagonize plant viruses and pathogenic bacteria, and Zhu et al. (2023) reported that a strain of *Cyberlindnera* can also inhibit *Botrytis cinerea* [49,50]. These observations provide candidate taxa for future isolation and functional characterization but do not establish their beneficial or pathogenic roles in *A. lappa*.

### 4.3. Variation in Soil Enzyme Activities in Response to Root Rot Infection in A. lappa

The primary active constituents of *A. lappa* include volatile oils (e.g., dihydrocostunolide, 12-methoxydihydrocostunolide), costunolide, dihydrocostus lactone, betulinic acids, and α-cyclocostunolide [51]. Soil enzymes influence the growth, secondary metabolism and other physiological processes of *A. lappa*, and they are crucial for soil carbon cycling, nutrient acquisition by plants, microbial activity, and overall soil health assessment [52,53,54,55]. In this study, compared with HLS, RRLS showed significantly higher cellulase and sucrase activities, whereas amylase, β-glucosidase, and β-xylosidase activities did not differ significantly. Laccase activity was significantly lower in RRLS. This may be attributed to the release of abundant nutrients during the early stages of the disease, which stimulates soil enzyme activity. In contrast, laccase activity exhibited a different trend. Because laccase production is regulated by complex microbial metabolic processes, its decreased activity may reflect shifts in microbial community composition or substrate availability rather than a simple reduction in secondary metabolism. The significantly lower laccase activity observed in RRLS compared to HLS may reflect changes in the microbial community and/or the availability of phenolic and lignin-derived substrates in the rhizosphere. Soil laccases are predominantly associated with microbial processes and are produced by various fungi and bacteria, where they participate in the transformation of lignin, phenolic compounds, and other components of soil organic matter [56,57]. Moreover, laccase activity can vary with microbial interactions and substrate availability [58]. Therefore, the reduced laccase activity in RRLS may represent a consequence of altered rhizosphere microbial composition or substrate conditions associated with root rot [59].

When root rot occurs, the cellulose within the root xylem is degraded by cellulase, which may account for the observed increase in cellulase activity [60,61]. The higher cellulase activity observed in RRLS may be consistent with increased degradation of cellulose-containing plant residues or damaged root tissues. However, the present study cannot determine whether altered cellulase activity preceded disease development or resulted from enhanced decomposition following root damage. Concurrently, the decomposition of plant tissues releases nutrients, leading to increased activities of other enzymes, such as amylase and sucrase. However, Zhang et al. (2023) reported reduced sucrase activity in root-rot-affected soils [62].

Zhao et al. (2024) observed that the activities of β-glucosidase and N-acetyl-glucosaminidase were lower in root-rot-infected soils compared to healthy soils, with no significant differences in other enzyme activities. Similarly, the study by Li et al. (2023a) on blueberry root rot indicated that antioxidant enzyme activities were lower in diseased soils than in those treated with biocontrol bacteria [63,64]. Our results differ from these previous findings, which may be due to the fact that we examined plants and soils at the initial stage of disease development. Studies have demonstrated that treating soybean root rot with the biocontrol strain HSY21 effectively reduces the activities of cellulase, β-glucosidase, and other enzymes associated with pathogens, suggesting higher enzymatic activity in root-rot-affected conditions, a result consistent with our findings [65]. Chatterton et al. (2021) reported no clear correlation between enzyme activity and root rot [66]. Thus, our results show both similarities and differences with earlier studies, which may be attributed to variations in plant species, disease progression stages, and sampling conditions.

As the commercial assay protocols required air-dried soil, the enzyme activities measured in this study represent standardized assay-based activities after sample processing rather than direct estimates of in situ extracellular enzyme activity. Although all samples underwent identical pretreatment, potential effects of air drying on absolute enzyme activity values cannot be excluded.

### 4.4. Coupling Relationships Among Nutrients, Enzymes, and Microbial Functional Shifts

The present study revealed strong coupling relationships among soil physicochemical properties, enzyme activities, and microbial functional profiles in the rhizosphere of *A. lappa*. Compared with HLS, RRLS showed significantly higher cellulase and saccharase activities, whereas amylase, β-glucosidase, and β-xylosidase activities did not differ significantly between the two loam-soil groups. These patterns likely reflect accelerated decomposition of root tissues during the early stage of root rot, which may release labile carbon substrates into the rhizosphere. Studies by Stock et al. (2019) and Geisseler and Horwath (2009) showed that the input of carbohydrate/cellulose (carbon) and amino acid/protein (nitrogen) substrates in the rhizosphere soil stimulates microbial activity and promotes the production of extracellular enzymes, thereby enhancing nutrient metabolic processes [67,68]. This is very similar to our conclusion in that RRLS exhibited higher concentrations of several nutrients together with altered carbon-cycle enzyme activities and higher predicted abundances of several microbial functional categories. These co-occurring patterns are consistent with increased substrate availability associated with root tissue decomposition. Predicted pathways related to carbohydrate metabolism, amino acid metabolism, energy metabolism, membrane transport, and glycan biosynthesis were more abundant in RRLS than in HLS. As these functions were inferred from marker-gene data, they should be interpreted as differences in predicted functional potential rather than evidence of pathway upregulation or metabolic activity. Consistently, functional prediction based on KEGG indicated that pathways related to carbohydrate metabolism, amino acid metabolism, energy metabolism, membrane transport, and glycan biosynthesis were enriched in RRLS. A correlation analysis further demonstrated that these functional categories were positively associated with soil nutrient levels and carbon-cycle enzyme activities, suggesting that nutrient enrichment and enzyme-mediated substrate turnover are associated with microbial functional responses. This is in line with previous studies showing that root-derived carbon inputs and organic matter decomposition can promote microbial carbon turnover and metabolic activity in the rhizosphere [69]. Taken together, differences in soil nutrient status and enzyme activities co-occurred with differences in predicted microbial functional profiles among the rhizosphere soil groups.

In a study on root-rot disease of *P. notoginseng*, KEGG prediction showed that pathways related to cell motility and membrane transport were more highly expressed in root-rot soil than in healthy soil, but pathways related to carbohydrate, energy metabolism, and biosynthesis of secondary metabolites were better expressed in healthy soil [70]. Our results partially support this study, as pathways related to cell motility and membrane transport show higher predicted relative abundance in root rot, but other results are not entirely the same. Cell motility and membrane transport may indicate a connection between bacterial movement and root colonization: pathogens move toward the roots and spread there after successfully infecting the pathogens [71]. In addition, membrane transport promotes the facultative growth of pathogens and other colonies by regulating the passage of soluble substances such as ions and small molecules through biological membranes, facilitating the successful infection of plant roots by pathogens through these two pathways [70].

The FUNGuild analysis further suggested ecological restructuring of fungal communities in root-rot-associated soils. RRLS showed higher relative abundance of predicted plant pathogen and saprotroph guilds, suggesting a microbial community shift toward decomposition-associated functional profiles. Conversely, healthy loam soils showed higher abundance of arbuscular mycorrhizal fungi and soil saprotrophs, potentially contributing to nutrient cycling and rhizosphere stability. These findings indicate that RRLS showed a higher relative representation of taxa assigned to pathogen- and saprotroph-associated guilds, whereas HLS contained a higher relative representation of some symbiotrophic guilds. These predicted guild differences indicate variation in the potential ecological composition of the fungal community but do not demonstrate pathogenic activity or a directional transition between ecological states. Given that the rhizosphere represents a hotspot of microbial activity and enzyme-mediated nutrient cycling [72], these changes may have important implications for plant–microbe interactions and disease development, although causal relationships require further validation. Research by Wang et al. (2022) [73] on *Sophora flavescens* showed that when symbiotrophs dominate in the soil, the plant’s resistance to disturbances strengthens. However, if the levels of pathotrophs and saprotrophs are higher than those of symbiotrophs, this easily leads to plant diseases. Consistent with Wang et al. (2022), HLS showed a relatively higher representation of some symbiotrophic fungal guilds, whereas RRLS showed a greater representation of taxa assigned to pathotrophic and saprotrophic guilds [73]. However, differences in host species, environmental conditions, and the predictive nature of FUNGuild assignments limit direct comparison between the two studies.

Soil texture also appeared to mediate rhizosphere microecology. Compared with healthy loam soils, healthy clay soils exhibited lower nutrient availability, reduced carbon-cycle enzyme activities, and weaker microbial functional enrichment. Although clay soils are generally considered to possess stronger nutrient-retention capacity, rhizosphere nutrient status is additionally shaped by microbial mineralization, root activity, and aeration. The compact structure and lower oxygen diffusion of clay soils may restrict microbial decomposition and root metabolic activity, thereby reducing microbial diversity and functional potential in the rhizosphere. The study by Zheng et al. (2021) on tobacco soil texture, plant-root nutrition, and microbial activity showed that clay has a lower capacity to supply nitrogen and fewer bacterial communities compared to loam, which is very similar to our results [74].

The study by Xia et al. (2020) on soil texture showed that coarse-textured soils provide more macropores than fine-textured soils and contain less water than fine-textured soils, thereby reducing the rate of resource diffusion/bacterial migration through pores [75]. Under moist conditions, the distribution of resources (such as oxygen) in coarse-textured soils is expected to be more heterogeneous than in fine-textured soils, and the physical activity range of bacteria is larger. This may explain why bacterial diversity is higher in coarse-textured soils. Previous studies have shown that coarse-textured soils promote bacterial richness under moist conditions [76]. We speculate that in clay, there are smaller pores that restrict the free movement of bacteria and fungi, which may also be the reason why fewer bacteria and fungi are observed. These mechanisms remain plausible explanations rather than experimentally demonstrated causes of the HLS–HCS differences because moisture, bulk density, parent material, and management history were not quantified.

## 5. Conclusions

This study demonstrates that root rot occurrence in *A. lappa* is associated with coordinated alterations in rhizosphere soil properties, extracellular enzyme activities, microbial community composition, and predicted functional potential. The integration of biogeochemical and microbial analyses reveals that root-rot-associated soils represent a distinct ecological state characterized by changes in nutrient cycling and microbial functional profiles, highlighting the importance of plant–soil–microbe interactions in understanding rhizosphere responses during disease development.

However, the present study was based on field observations, and the causal relationships between soil ecosystem changes and root rot development remain unresolved. Future studies combining controlled pathogen inoculation, long-term field monitoring, and functional validation of key microbial taxa are needed to clarify whether these ecological changes contribute to disease susceptibility, result from disease progression, or represent feedback responses within the rhizosphere ecosystem.

## Figures and Tables

**Table 1:** The alpha diversity of microbial communities in different *Aucklandia lappa* habitats (analysis via one-way ANOVA and Tukey HSD multiple-comparison test. ns: no significant difference; * *p* < 0.05; ** *p* < 0.01; *** *p* < 0.001; **** *p* < 0.0001). RRLS and HCS both show significant differences from HLS. Values are presented as mean ± SD (*n* = 6 independent biological replicates per soil category).

Group	Bacterial Community			Fungal Community		
	Chao1	Simpson	Shannon	Chao1	Simpson	Shannon
HLS	3759.27 ± 480.26	0.026 ± 0.027	5.76 ± 0.80	1675.43 ± 425.39	0.050 ± 0.030	4.31 ± 0.66
RRLS	4029.33 ± 255.16 *	0.006 ± 0.0003 ns	6.42 ± 0.03 ns	1676.77 ± 96.23 ns	0.034 ± 0.002 ns	4.42 ± 0.05 ns
HCS	4000.58 ± 201.84 ns	0.011 ± 0.0009 ns	5.99 ± 0.03 ns	899.54 ± 119.53 ****	0.162 ± 0.021 ***	2.83 ± 0.20 ****

## Data Availability

The raw 16S rRNA gene and ITS amplicon sequencing data generated in this study are available in the NCBI Sequence Read Archive under BioProject accession PRJNA1353648. The supporting data used for the analyses are provided in the article and its Supplementary Materials.

## Supplementary Materials

The following supporting information can be downloaded at: https://www.mdpi.com/article/10.3390/microorganisms14092049/s1. Figure S1: Venn diagram of OTUs obtained from sequencing. (a) bacteria; (b) fungus; Figure S2: Correlation analysis between predicted bacterial functions and physicochemical factors, red indicates positive correlation, blue indicates negative correlation. Figure S3: Rarefaction curve of the bacterial microbiome sequencing; Figure S4: Rarefaction curve of the fungal microbiome sequencing; Table S1: Collection information of *Aucklandia lappa* samples in current study; Table S2: Physical and chemical properties of different *Aucklandia lappa* samples; Table S3: Annotation table of bacterial OTUs for each soil sample (1: present; 0: absent); Table S4: Annotation table of fungal OTUs for each soil sample (1: present; 0: absent); Table S5: Alpha diversity index in each soil sample (ITS: Fungi; 16S: Bacterial); Table S6: Relative abundance of bacterial phyla in *A. lappa* soil; Table S7: Relative abundance of bacterial genera in *A. lappa* soil; Table S8: Relative abundance of fungal phyla in *A. lappa* soil; Table S9: Relative abundance of fungal genera in *A. lappa* soil; Table S10: The carbon content and enzyme activities in different *Aucklandia lappa* habitats. (Using one-way ANOVA and Tukey HSD multiple comparison test, ns: no significant difference; RRLS and HCS both conduct significance difference analysis with HLS.
