# Supplementary material for: Rhizosphere Microbial and Biogeochemical Differences Associated with Root Rot Occurrence in Aucklandia lappa
Source: Microorganisms. 2026 Sep 14;14(9):2049. doi: 10.3390/microorganisms14092049 (PMC13608959; doi:10.3390/microorganisms14092049)
Supplement: Supplementary file 1 [file microorganisms-14-02049-s001.zip › Supplementary Table S1.pdf]

Supplementary Table S1. Collection information of *Aucklandia lappa* samples in current study

Sample	Health condition	Sand (%)	Silt (%)	Clay (%)	soil texture	Groups	Longitude and latitude
A1	Health	37.6	48.8	13.6	Loam	HLS	108.772229E,31.512417N
A2	Health	42.4	44.7	12.9	Loam	HLS	108.772062E,31.512093N
A3	Health	39.1	47.7	13.2	Loam	HLS	108.767910E,31.513370N
A4	Health	46.3	39.1	14.6	Loam	HLS	108.765822E,31.513675N
A5	Health	41.1	47.2	11.7	Loam	HLS	108.765748E,31.514006N
A6	Health	42.9	46.2	10.9	Loam	HLS	108.765947E,31.514244N
A7	Root rot	44.7	41.1	14.2	Loam	RRLS	108.759115E,31.515099N
A8	Root rot	46.8	39.7	13.5	Loam	RRLS	108.753685E,31.511982N
A9	Root rot	39.3	46.3	14.4	Loam	RRLS	108.748791E,31.509501N
A10	Root rot	36.1	51.0	12.9	Loam	RRLS	108.723076E,31.503904N
A11	Root rot	42.5	47.3	10.2	Loam	RRLS	108.739241E,31.507279N
A12	Root rot	45.7	44.6	9.7	Loam	RRLS	108.727895E,31.509571N
A13	Health	12.7	28.9	58.4	Clay	HCS	108.724117E,31.509135N
A14	Health	14.8	29.1	56.1	Clay	HCS	108.730462E,31.506803N
A15	Health	13.3	26.9	59.8	Clay	HCS	108.737842E,31.510749N
A16	Health	12.3	25.2	62.5	Clay	HCS	108.737651E,31.510274N
A17	Health	15.9	19.4	64.7	Clay	HCS	108.731074E,31.507331N
A18	Health	15.2	25.6	59.2	Clay	HCS	108.748612E,31.511046N
