# Supplementary material for: Rhizosphere Microbial and Biogeochemical Differences Associated with Root Rot Occurrence in Aucklandia lappa
Source: Microorganisms. 2026 Sep 14;14(9):2049. doi: 10.3390/microorganisms14092049 (PMC13608959; doi:10.3390/microorganisms14092049)
Supplement: Supplementary file 1 [file microorganisms-14-02049-s001.zip › Supplementary Table S10.pdf]

Supplementary Table S10. The carbon content and enzyme activities in different *Aucklandia lappa* habitats. (Using one-way ANOVA and Tukey HSD multiple comparison test, ns: no significant difference; RRLS and HCS both conduct significance difference analysis with HLS.

Groups	Total carbon (mg/g)	Activities of soil enzymes (U/g)					
		Saccharase	Amylase	β-Xylosidase	β-Glucosidase	Cellulase	Laccase
HLS	24.67±6.93	45.04±9.04	19.53±7.55	7.41±1.83	25.95±9.08	0.042±0.018	15.58±2.98
RRLS	34.56±5.89, P=0.0102	177.97±26.23, P<0.0001	23.95±6.65ns	9.17±2.12ns	30.12±6.63ns	0.065±0.013, P=0.0139	5.91±2.89, P<0.0001
HCS	4.43±0.98, P<0.0001	18.55±9.00, P=0.0221	7.53±1.72, P=0.0057	2.37±0.83, P=0.0002	12.09±3.55, P=0.0058	0.009±0.004, P=0.0010	6.90±2.58, P=0.0002
