# Supplementary material for: Rhizosphere Microbial and Biogeochemical Differences Associated with Root Rot Occurrence in Aucklandia lappa
Source: Microorganisms. 2026 Sep 14;14(9):2049. doi: 10.3390/microorganisms14092049 (PMC13608959; doi:10.3390/microorganisms14092049)
Supplement: Supplementary file 1 [file microorganisms-14-02049-s001.zip › Supplementary Table S2.pdf]

Supplementary Table S2. Physical and chemical properties of different *Aucklandia lappa* samples.

Sample	PH	Available nitrogen	Available phosphorus	Available potassium	Organic matter
A1	5.93	323.556	32.242	902.754	23.112
A2	6.18	234.264	66.448	323.931	29.164
A3	5.97	330.472	45.293	282.633	72.213
A4	5.61	315.992	64.126	834.043	93.137
A5	6.11	274.617	34.638	364.306	63.263
A6	6.05	200.793	17.081	584.835	35.182
A7	5.80	370.147	101.015	1475.469	97.814
A8	5.82	385.286	109.636	1536.732	103.729
A9	5.78	378.962	116.172	1492.682	95.738
A10	5.90	361.473	103.947	1439.065	92.651
A11	5.88	349.609	95.726	1425.792	99.462
A12	5.93	369.628	89.674	1518.026	106.382
A13	6.28	87.508	33.531	215.161	25.666
A14	6.15	69.419	26.772	190.281	24.716
A15	6.22	74.285	38.912	241.793	26.146
A16	6.19	91.872	42.659	257.907	26.673
A17	6.31	101.643	36.134	205.174	24.958
A18	6.17	89.494	28.537	186.619	22.795
